# Asthma and the Diversity of Fungal Spores in Air

**DOI:** 10.1371/journal.ppat.1003371

**Published:** 2013-06-06

**Authors:** Anne Pringle

**Affiliations:** Organismic and Evolutionary Biology, Harvard University, Cambridge, Massachusetts, United States of America; Duke University Medical Center, United States of America

The diversity of fungal spores in air is vast ([1], [2], Figure 1), but research on asthma focuses on a handful of easily identified, culturable species. Ecologists are developing new tools to probe communities and identify the full complement of fungi in habitats. These tools may enable identification of novel asthma triggers, but scientists involved in public health or medicine rarely interact with mycologists focused on ecology. With this primer, my aim is to facilitate communication by providing doctors with a basic, modern guide to spores, by teaching mycologists the essential facts of asthma, and by providing patients with a succinct summary of what is known about spores and asthma. By highlighting the use of emerging metagenomics technologies in ecology, I intend to illustrate how these tools might be used to more thoroughly understand the potential diversity of fungi involved in asthma.

**Figure 1:**
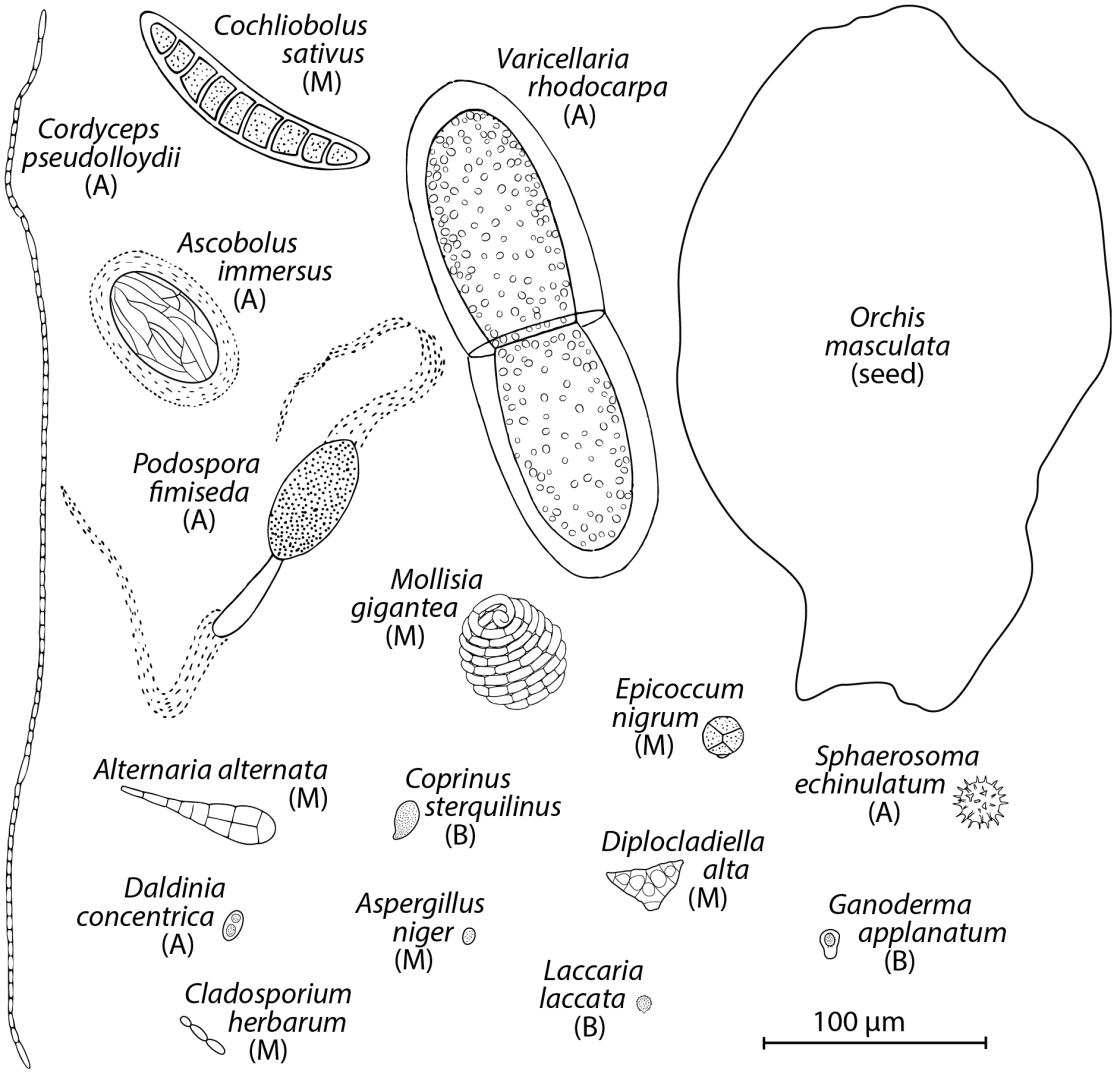
Drawings of spores found in air. Some are common targets of research on asthma (*Alternaria alternata*), while many are not (*Laccaria laccata*, *Epicoccum nigrum*). A drawing of a very small “dust” seed (*Orchis masculata*) is provided for comparison. A = ascospore, B = basidiospore, M = mitospore. Figure adapted from [30].

## What Is Asthma?

Asthma is a complex disease without a single cause, defined by its symptoms [3]. Wheezing, chest tightness, and breathlessness are triggered by airway inflammation. The natural histories of asthma are diverse and involve both genetic and environmental parameters. For example, children with variants of the *ADAM33* gene are at risk for asthma [4], while attendance at day care in the first six months of life and the presence of older siblings appear to protect against asthma [5]. Allergies are intimately associated with asthma. Asthma can result from direct inhalation of allergens, but atopic asthmas are caused by the body's interaction with allergens outside of the lungs. Asthma is a public health burden, and the prevalence of asthma is increasing [6]. In countries including Australia, Canada, and Peru, over a quarter of 13 to 14-year-old children report wheezing [7], and in 2007, medical expenses associated with asthma cost the United States US$50.1 billion (www.cdc.gov/asthma/). Asthma may count for one in every 250 deaths worldwide [7]. However, asthma rates vary substantially with geography, and for example are very low in Indonesia and Romania [7]. The severity of asthma among patients also varies substantially; some manage asthma with inhalers, while others are frequently hospitalized. The literature suggests a role for fungi in the etiology of asthma, and in medicine SAFS is a diagnosis used to delineate “Severe Asthma with Fungal Sensitization,” but whether the entire diversity of fungi is involved in causing asthma is unknown.

## Why Do Fungi Make Spores? And a Guide to Terminology

The jargon associated with fungal spores is fantastically diverse, but basic aspects of identification hinge on reproductive mode and identification to phylum. Most species make both sexual and asexual spores. Sexual spores are meiospores and asexual spores are mitospores. Sexual spores of species in the phylum Ascomycota are ascospores, and sexual spores of the phylum Basidiomycota are basidiospores. Asexual spores of either group are often termed conidia, although asexual spores have other names as well. Fungi are no longer classified as “deuteromycetes” or discussed as “imperfect” because molecular approaches have confirmed deuteromycetes as lineages of Ascomycetes or Basidiomycetes [8]; however, the terms are still found in current asthma literature. Other fungal phyla make spores, including for example the Glomeromycota and Chytridiomycota, but they appear to be a minority component of the biodiversity of air [1], [2].

Fungi make spores to disperse to new habitats. A single species often makes multiple kinds of spores; for example, over the course its life cycle, a rust pathogen may make one sexual and up to five different types of asexual spores. The numbers involved are staggering. A single mushroom of the giant puffball *Calvatia gigantea* discharges 7 trillion spores, and the smaller *Coprinus sterquilinus* discharges 100 million, though *C. sterquilinus* often grows as clusters of multiple mushrooms [9]. These species are Basidiomycetes. A typical estimate for an Ascomycete is 10 million spores per plant, taken from the plant pathogen *A. alternata* [10]. Global average abundance is estimated at 1 µg per m^3^ of air [11]. Statistics on the average number of spores a human breathes are difficult to find, perhaps because spores will be far more abundant in particular habitats (for example, enclosed, damp, poorly ventilated spaces [12]), and fungi will sporulate at greater rates at particular times of year (for example, late summer in temperate climates [13]). One potentially conservative estimate is between 60–60,000 spores per day; the higher number is relevant to moldy buildings or the outdoors [14]. However, no matter the location or season, humans interact with fungi and spores on a daily basis.

## Do Fungal Spores Cause Asthma?

A wealth of correlative evidence suggests asthma is associated with fungi and triggered by elevated numbers of fungal spores in the environment [15]. Most intriguing are reports of “thunderstorm asthma.” In a now classic study from the United Kingdom, an outbreak of acute asthma was linked to increases in *Didymella exitialis* ascospores and *Sporobolomyces* basidiospores associated with a severe weather event [16]. Thunderstorms are associated with spore plumes: when spore concentrations increase dramatically over a short period of time, for example from 20,000 spores/m^3^ to over 170,000 spores/m^3^ in 2 h [17]. However, other sources consider pollen or pollution as causes of thunderstorm asthma [18]. Transoceanic and transcontinental dust events move large numbers of spores across vast distances and have the potential to impact public health [19], and similar correlative evidence links dust blown off the Sahara with pediatric emergency room admissions on the island of Trinidad [20]. However, other studies have found no association between asthma and the fungi in Saharan dust [21]. Indoors, asthma symptoms are strongly correlated with exposure to fungi [12], [15], for example *Alternaria alternata* in the home [22], but for reasons which may have to do with genetics or circumstances, not all persons are susceptible. Moreover, despite the focus on spores, there is evidence to suggest hyphal fragments will be far more common than spores in indoor environments, and will also play a role in exposure to fungi [23]. Hyphal fragments are typically smaller than spores (<1 µm) and may penetrate lungs more effectively. Correlations between spores and asthma symptoms may reflect a different correlation between spores and hyphal fragments.

When spores trigger asthma, it's not clear if the spores are provoking an immune response or functioning as small particles that penetrate and irritate the lungs; both mechanisms may be relevant. Immune responses may be caused by allergens bound to the surface of a spore, or perhaps more commonly, by allergens appearing after a spore germinates, when a fungus is growing. The ability of any particular species to provoke asthma may depend on environmental parameters; for example, genes encoding known allergens are more highly expressed in conidia of *Aspergillus fumigatus* grown at lower temperatures [24].

## Which Species Are Associated with Asthma?

Research on asthma and fungi is often inconsistent, and public health data focus on very few species. A typical count of spores lists *Alternaria*, *Cladosporium*, *Ganoderma*, “other Basidiomycetes,” and “Ascomycetes” (because *Alternaria* and *Cladosporium* are considered as deuteromycetes). The data are being driven by what can be identified visually and counted, and not by the true species richness of air. For example, Packe and Ayres [16] give no data for the ascospores and basidiospores of fungi other than *Cladosporium*, *D. exitialis*, and *Sporobolomyces*, and the species of *Cladosporium* and *Sporobolomyces* are not identified. But they had more reason than most authors to focus on this subset of species: workers at the Rothamsted agricultural research station had identified *D. exitialis* as particularly abundant in late summer in England and suspected it as a trigger of their own asthma, a suspicion supported by skin tests [25]. But the fungi encompass several million species and all of these make spores, many (even most) of which will be found in the air humans breathe. Different species or spore types may have different surface properties or internal metabolisms, and different potentials as causes of asthma. While logic would dictate a systematic search for triggers grounded in what's most abundant in the environment [1], [2], instead research has focused on easily cultured fungi with distinct spore shapes. Sometimes it's a good match: *Alternaria* and *Cladosporium* are easily cultured and abundant in indoor environments [8], while other targets of research, including *Aspergillus*, *Trichophyton*, and *Malassezia*, are commonly found in association with humans. But often there's a disconnect: Basidiomycetes are rarely considered as aeroallergens [26], even though groups including the Agaricomycetes, or mushrooms, are abundant in temperate, terrestrial atmospheres [1].

## If Identification to Species Matters, Will New Tools Provide Needed Data?

Yes. While establishing a cause and effect between spores and asthma may remain a challenge, metagenomic technologies will establish correlations between the diversity of fungi and asthma more effectively. Ecologists faced a similar dilemma at the start of the molecular revolution: the biodiversity of what can be seen and counted is different from true biodiversity [27]. Ecologists used to count mushrooms to measure numbers of species, but direct sequencing of environmental samples suggests in most ecosystems, what can be counted by hand is not the same as what's growing. Unfortunately, the worlds of medicine and ecology rarely intersect, but doctors and aerobiologists can capitalize on the experiences of ecologists [2], [27] to probe and document the true diversity of fungi in the homes and lungs of asthmatic patients, and for example test for what's found in the air just before or after a thunderstorm. Metagenomics data are likely to provide a very different understanding of the potential diversity of spores involved in asthma (compare [2], [16]). Perhaps profiles of lungs will reveal basidiospores as more common than previously thought, or identify a currently undescribed species as particularly abundant [27]. That undescribed species may emerge as a novel target for research.

We can't be sure identification of the diversity of fungi in a patient's lungs will enable more effective treatments, but fungi are not a monolithic entity, and it's likely some groups or species will be more able to trigger asthma than others. Enumerating diversity may enable identification of currently uncultured species with relevant triggers. It's likely differences in the niches of species will influence genetic architectures [28] and metabolic profiles, and in these cases identification to species may be critical to effective treatment. Effective treatment may involve ecology: perhaps a home can be engineered to avoid the growth of a particular species with a defined niche.

As disease prevalence continues to rise, enabling research on the roles of fungi in asthma makes sense. At the moment, there are as many questions as answers. Moreover, humans appear to be reshaping the ecology of the kingdom: elevated CO_2_ concentrations appear to stimulate sporulation [10], [29], and climate change appears to influence the timing of sporulation [13]. A current hypothesis suggests a connection between global change and the rise of asthma and allergies [10]. There has never been a more relevant moment for interactions between medicine and ecology.
